# Supplementary material for: Hierarchical Reconstruction of High-Resolution 3D Models of Large Chromosomes
Source: Sci Rep. 2019 Mar 21;9:4971. doi: 10.1038/s41598-019-41369-w (PMC6428844; doi:10.1038/s41598-019-41369-w)
Supplement: Supplementary file 1 — Supplementary Information [file 41598_2019_41369_MOESM1_ESM.docx]

**Supplementary Information**

**Hierarchical Reconstruction of High-Resolution 3D Models of Large Chromosomes**

Tuan Trieu, Oluwatosin Oluwadare, and Jianlin Cheng^*^

Department of Electrical Engineering and Computer Science, University of Missouri-Columbia, MO 65211, USA

*chengji@missouri.edu


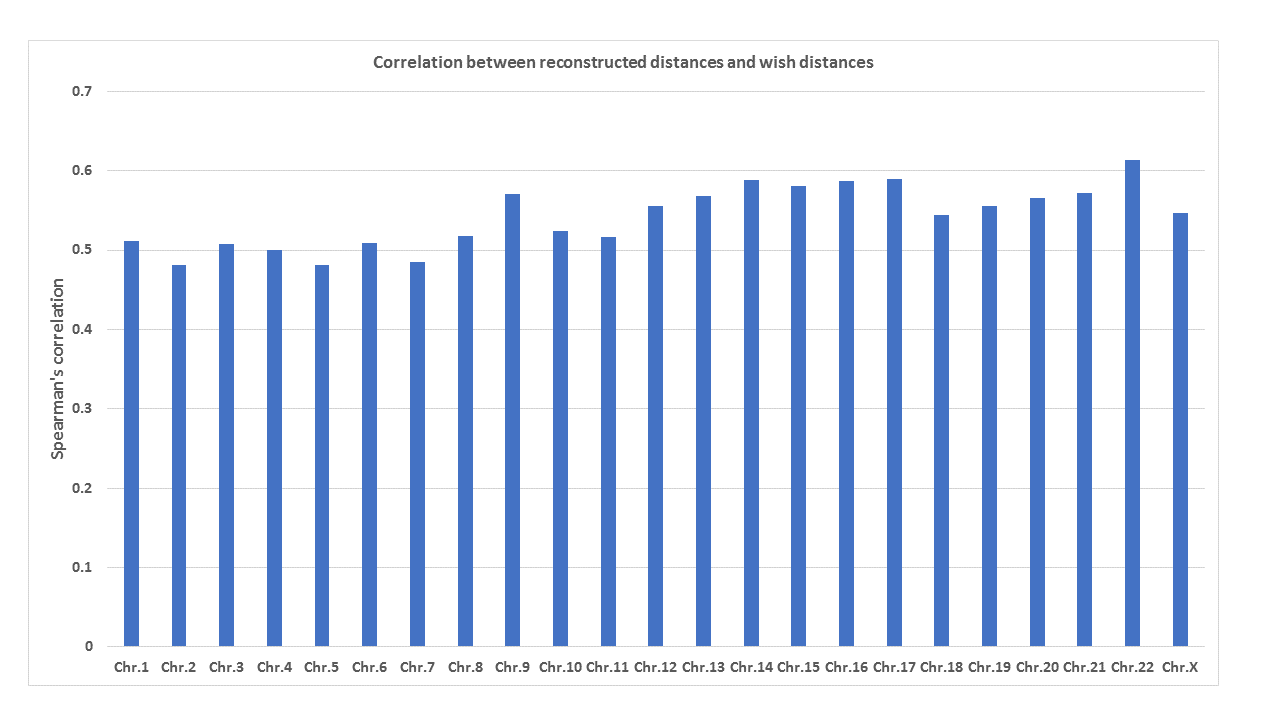
**Supplementary Figure S1**. Correlation between reconstructed distances and expected distances for all 23 pairs of human chromosomes.

**Supplementary Figure S2.** Correlation between the contact maps derived from chromosomal models and the input Hi-C data contact map for each chromosome at increasing IF cut-off threshold 0,1,3,5,7,10. Contacts below each threshold are cut-off and not used in the Spearman correlation calculation for each chromosome. An increase in spearman correlation as only high IF
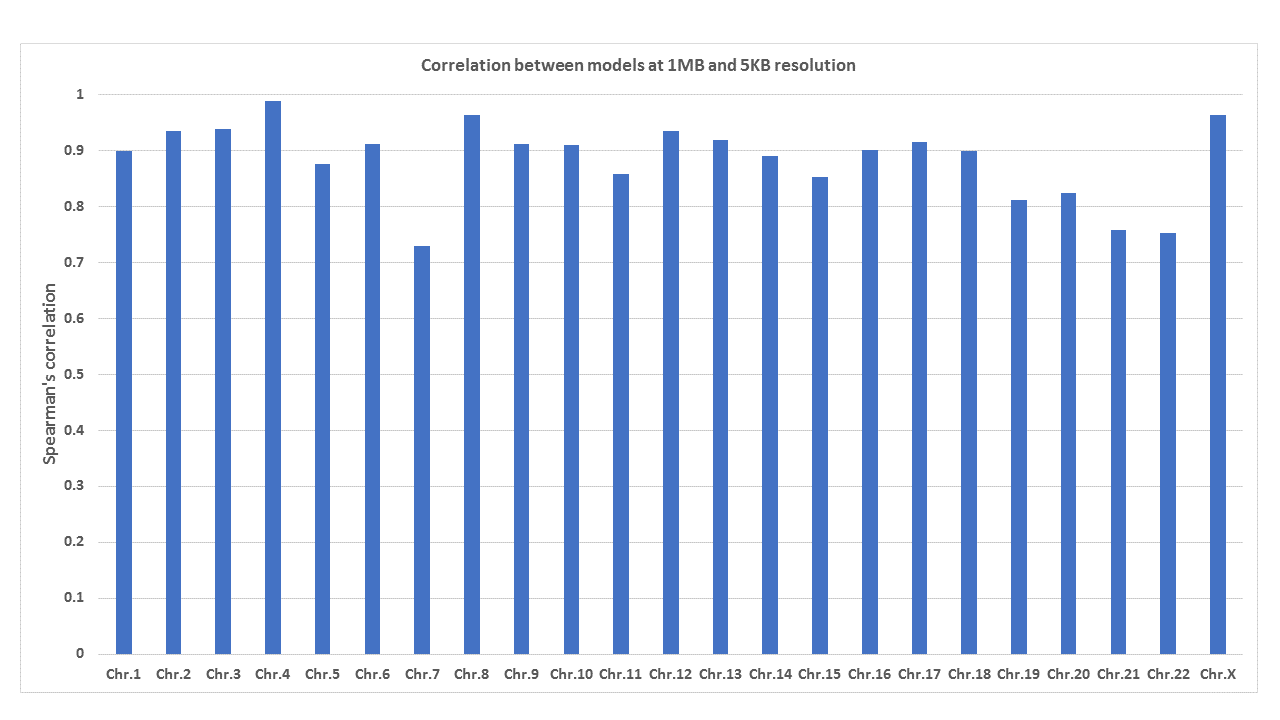
contacts are used and low IF contacts are removed.

**Supplementary Figure S3**. Spearman's correlation between distances from models at 1MB and 5KB resolution. Models at 5KB resolution are consistent with models at 1MB resolution.

**Supplementary Figure S4**. Accuray of Hierarchical3DGenome and miniMDS for each chromosome at 5KB.

**Supplementary Table S1** – Distance within TAD and between adjacent TAD in chromosome models.

Validation of the satisfaction of the input TADs in the chromosome models by computing the average Euclidean distance of the bins within a TAD and those between two adjacent TADs for all the chromosomes.

| Chromosome | Average Distance within TAD | Average Distance between  TADs |
| --- | --- | --- |
| 1 | 18.8816 | 66.7513 |
| 2 | 19.3875 | 66.1513 |
| 3 | 15.8373 | 64.7524 |
| 4 | 13.1504 | 57.5756 |
| 5 | 13.9163 | 56.1272 |
| 6 | 15.4191 | 67.4322 |
| 7 | 22.0771 | 64.8718 |
| 8 | 18.2871 | 66.8777 |
| 9 | 18.9194 | 76.4743 |
| 10 | 21.3297 | 67.8757 |
| 11 | 14.1123 | 59.4074 |
| 12 | 17.4266 | 65.0315 |
| 13 | 17.22 | 69.0305 |
| 14 | 11.1328 | 62.408 |
| 15 | 15.4691 | 69.5117 |
| 16 | 27.9239 | 79.306 |
| 17 | 12.5207 | 42.4048 |
| 18 | 18.434 | 73.2308 |
| 19 | 15.039 | 49.8911 |
| 20 | 18.4269 | 68.0942 |
| 21 | 20.4797 | 62.088 |
| 22 | 11.8237 | 48.7793 |
| X | 18.6178 | 78.1339 |
